# Toward the Synthesis and Biological Screening of a Cyclotetrapeptide from Marine Bacteria

**DOI:** 10.3390/md9010071

**Published:** 2010-12-30

**Authors:** Rajiv Dahiya, Hemendra Gautam

**Affiliations:** Department of Pharmaceutical Chemistry, NRI Institute of Pharmacy, Bhopal 462 021, Madhya Pradesh, India; E-Mail: hemendra_gautam2002@yahoo.com

**Keywords:** natural product synthesis, marine bacteria, cyclic tetrapeptide, biological activity, *Halisarca ectofibrosa*, *Diginea* sp

## Abstract

The first synthesis of a naturally occurring tetrapeptide *cyclo*-(isoleucyl-prolyl-leucyl- alanyl) has been achieved using a solution-phase technique via coupling of dipeptide segments Boc-l-Pro-l-Leu-OH and l-Ala-l-Ile-OMe. Deprotection of the linear tetrapeptide unit and its subsequent cyclization gave a cyclopeptide, identical in all aspects to the naturally occurring compound. Bioactivity results indicated the antifungal and antihelmintic potential of the synthesized peptide against pathogenic dermatophytes and earthworms.

## 1. Introduction

Naturally occurring cyclic peptides have generated much interest in recent years due to their intriguing chemical structures and potent biological activity. They show therapeutic potential as a result of greater resistance to *in vivo* enzymatic degradation as well as greater bioavailability than non-cyclic analogs. Cyclooligopeptides, especially medium ring-sized peptides bearing aliphatic amino acid units, have been reported from diverse marine sources, including marine microorganisms [1–5]. These cyclic congeners derived from marine microorganisms exhibit a range of pharmacological activities, including antimicrobial activity [6], cytotoxicity [7,8], anti-dinoflagellate activity [9] and inhibitory activity against enzyme sortase B [10]. A natural cyclotetrapeptide *cyclo*-(isoleucyl-prolyl-leucyl- alanyl) has been isolated from the marine bacteria *Pseudomonas* sp. and *Pseudoalteromonas* sp., associated with the seaweed *Diginea* sp. and the sponge *Halisarca ectofibrosa* and the peptide’s structure was elucidated on basis of LC-MS/2D NMR data [11].

Keeping in view the wide array of bioactivities possessed by natural cyclooligopeptides [12,13] and in continuation of our previous investigations on peptides [14–22], an attempt was made toward the synthesis of a natural cyclic tetrapeptide (**4**) employing a solution-phase synthesis method. In addition, the synthesized product was further subjected to anthelmintic and antimicrobial activity studies.

## 2. Results and Discussion

**Scheme 1:**
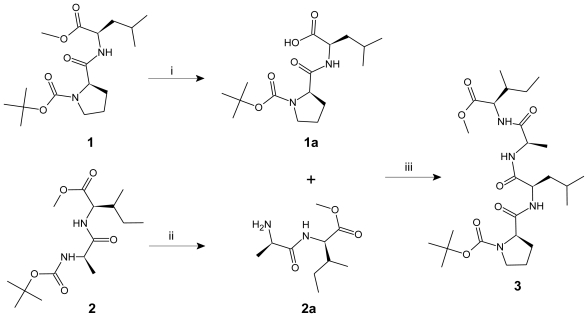

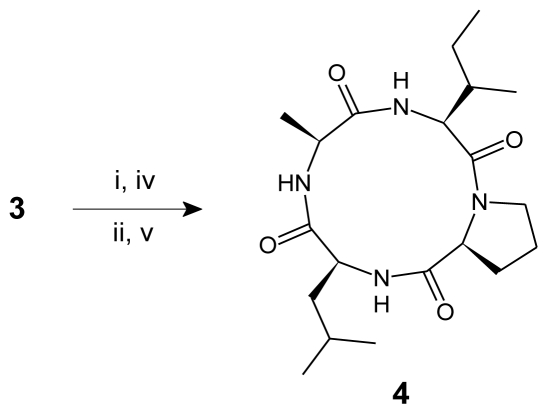
Synthesis of cyclotetrapeptide **4**.

The title compound **4** was synthesized by the method shown in Scheme 1. In order to carry out the synthesis of *cyclo*-(isoleucyl-prolyl-leucyl-alanyl), the molecule was split into two dipeptide units: Boc-l-Pro-l-Leu-OMe (**1**) and Boc-l-Ala-l-Ile-OMe (**2**). The dipeptides were prepared by coupling Boc-amino acids *viz.* Boc-l-Pro-OH and Boc-l-Ala-OH with respective amino acid methyl ester hydrochlorides like l-Leu-OMe.HCl and l-Ile-OMe.HCl using 1-ethyl-3-(3-dimethylaminopropyl)carbodiimide hydrochloride (EDC.HCl) as coupling agent and pyridine as base [23]. The ester group of dipeptide **1** was removed with lithium hydroxide (LiOH) and deprotected peptide **1a** was coupled with dipeptide **2a** deprotected at the amino terminal using trifluoroacetic acid (TFA), to get the linear tetrapeptide unit Boc-l-Pro-l-Leu-l-Ala-l-Ile-OMe (**3**). Finally, cyclization of the linear tetrapeptide **3** was done by the pentafluorophenyl ester method to obtain the cyclooligopeptide *cyclo*-(isoleucyl-prolyl-leucyl-alanyl) (**4**). Structures of newly synthesized cyclopeptide and intermediate peptides were elucidated by spectral/elemental analysis (Tables 1 and 2).

Synthesis of the natural cyclotetrapeptide **4** was completed successfully from the linear precursor and *N*-methylmorpholine (NMM) was proven to be an effective base for cyclizing linear tetrapeptide segment. Disappearance of absorption bands at 1745, 1272 cm^−1^ and 1392, 1375 cm^−1^ (C=O_str_ and C–O_str_, methyl ester group and C–H_bend_, *tert*-Butyl group) in FT-IR spectrum of **4** clearly indicated cyclization of the linear tetrapeptide unit. This fact was further supported by the disappearance in the ^1^H NMR spectrum of **4** of two singlets at *δ* 1.48 and *δ* 3.49 corresponding to protons of *tert*-Butyl and methyl ester groups of **3**. Four signals between *δ* 4.26–3.61 in the proton spectrum of **4** suggested a peptidic structure for the synthesized product, with these signals being attributable to the α-protons of all amino acid units. The ^1^H NMR spectrum of the cyclized product showed the presence of three broad singlets between *δ* 9.37–7.76 corresponding to the imino protons of the isoleucine, leucine and alanine moieties, the remaining amino acid being the proline unit, indicating similarity of the structure of the newly synthesized cyclotetrapeptide with the natural molecule. The NMR data of the synthetic cyclotetrapeptide are identical with those of the natural product within the error range of *δ* 0.06 (^1^H) and 1 (^13^C), respectively. Moreover, ^1^H/^13^C NMR spectra of the cyclized product **4** showed characteristic peaks confirming the presence of all the 34 protons and 20 carbon atoms. Presence of pseudomolecular ion peak at *m*/*z* 395.4 corresponding to the molecular formula C_20_H_34_N_4_O_4_ in the mass spectrum of **4**, along with other fragment ion peaks resulting from cleavage at “Leu-Pro”, “Ile-Ala” and “Ala-Leu” amide bonds, showed the exact sequence of attachment of all the four amino acid units in the chain. In addition, elemental data analysis of **4** afforded values (±0.02) strictly in accordance with the molecular composition.

The newly synthesized cyclooligopeptide **4** exhibited potent anthelmintic activity at a concentration of 2 mg/mL in tween 80 (0.5%) and distilled water. Comparison of the anthelmintic activity data revealed that the cyclopeptide **4** displayed greater activity than it’s corresponding linear precursor **3** and the standard drug, mebendazole, against all three earthworm species *M. konkanensis*, *P. corethruses* and *E. eugeniea* (Table 3).

Analysis of the antimicrobial activity data indicated that cyclopeptide **4**, compared to the standard drug gatifloxacin, displayed moderate antibacterial activity against the Gram-negative bacteria *P. aeruginosa* and *K. pneumoniae*, and antifungal activity against pathogenic *C. albicans* with minimum inhibitory concentration (MIC) values of 6 μg/mL. Moreover, **4** displayed potent bioactivity against dermatophytes *M. audouinii* and *T. mentagrophytes* with MIC values of 6 μg/mL. However, **4** displayed no bioactivity against either Gram-positive bacteria or against the fungus *A. niger* (Table 4).

## 3. Experimental Section

Melting points were determined in open capillaries and are uncorrected. IR spectra were recorded on a Shimadzu 8700 FTIR spectrophotometer and ^1^H/^13^C NMR spectra were recorded on a Bruker AC NMR spectrometer (300 MHz) using deuterated chloroform as solvent and TMS as internal standard. The mass spectra were recorded on a JMS-DX 303 Mass spectrometer operating at 70 eV by ESIMS/MS. Optical rotation of synthesized peptide derivatives was measured on an automatic polarimeter at 25 °C using a sodium lamp. Elemental analysis of all compounds was performed on a Vario EL III elemental analyzer. Purity of all synthesized compounds was checked by TLC on precoated silica gel G plates.

### 3.1. Extraction and Isolation of Natural Cyclic Tetrapeptide

As reported earlier, a bacterial strain identified as a *Pseudomonas* sp. by 16S rRNA analysis was obtained from the Japanese seaweed *Diginea* sp., an alga which has a symbiotic relationship with dinoflagellates such as *Amphidinium* sp. The colonies inhibited the growth of other marine bacteria when grown on seawater based agar medium. Purification by SiO_2_ column chromatography and reversed-phase HPLC of the CHCl_3_-MeOH soluble fractions of the culture supernatant gave peptide-containing fractions that were analyzed by ^1^H NMR and by LC-MS. Extensive 2D NMR analysis of the individual cyclopeptide components confirmed the isolation of the novel cyclotetrapeptide *cyclo*-(isoleucyl-prolyl-leucyl-alanyl) in addition to other previously characterized peptides [11].

The culture broth (1.5 L) of a bacterial strain No. 27 (*Pseudomonas* sp.) was centrifuged, and the bacterial cells were extracted with CHCl_3_/MeOH (1:1, 500 mL). The combined extracts were concentrated, and the residue was partitioned with EtOAc and water. The EtOAc layer (152 mg) showed potent antimicrobial activity against an orange-colored unidentified bacterium associated with the same host (*Diginea* sp.). After further separation by Si gel chromatography and elution using a solvent gradient of hexanes/CHCl_3_/EtOAc/MeOH, the fraction eluted in EtOAc/MeOH (1:1) was subjected to reverse phase HPLC using a MeOH/H_2_O gradient from 50 to 100% MeOH and UV detection at 215 nm to give the desired cyclic tetrapeptide (2.0 mg).

### 3.2. General Procedure for the Preparation of Linear Dipeptide Fragments

l-Amino acid methyl ester hydrochloride (0.01 mol) was dissolved in CH_2_Cl_2_ (20 mL). Pyridine (1.61 mL, 0.021 mol) was added to the mixture at 0 °C and stirred for 15 min. Boc-l-amino acid (0.01 mol) was dissolved in CH_2_Cl_2_ (20 mL) followed by addition of EDC.HCl (1.92 g, 0.01 mol) and HOBt (1.34 g, 0.01 mol). The resulting mixture was added to the above solution with constant shaking and stirring was continued for 24 h. The reaction mixture was filtered and the residue was washed with CH_2_Cl_2_ (30 mL) and added to the filtrate. The filtrate was washed with 5% NaHCO_3_ and saturated NaCl solutions. The organic layer was dried over anhydrous Na_2_SO_4_, filtered and evaporated in vacuum. The crude product was recrystallized from a mixture of chloroform and petroleum ether (b.p. 40–60 °C) followed by cooling at 0 °C to get the title compounds.

### 3.3. Procedure for the Synthesis of Linear Tetrapeptide Unit and Its Cyclization

l-Alanyl-l-isoleucine methyl ester **2a** (2.16 g, 0.01 mol) was dissolved in DMF (25 mL) and pyridine (0.021 mol) was added to the above solution in proportions. Finally, the reaction mixture was stirred for 30 minutes, while maintaining the temperature between 0–5 °C. Boc-l-prolyl-l-leucine **1a** (3.28 g, 0.01 mol) was dissolved in DMF (35 mL) and EDC.HCl (1.92 g, 0.01 mol) and HOBt (1.34 g, 0.01 mol) were added in proportions while stirring. Stirring was first done for 1 h at 0–5 °C and then further for 24 h at room temperature (RT). After the completion of the reaction, the reaction mixture was diluted with an equal amount of water. The precipitated solid was filtered, washed with water and recrystallized from a mixture of chloroform and petroleum ether (b.p. 40–60 °C), followed by cooling at 0 °C to get Boc-l-prolyl-l-leucyl-l-alanyl-l-isoleucine methyl ester **3**. The linear tetrapeptide unit **3** (2.63 g, 0.005 mol) was deprotected at the carboxyl terminal using lithium hydroxide (LiOH, 0.18 g, 0.0075 mol) to obtain Boc-l-prolyl-l-leucyl-l-alanyl-l-isoleucine-OH. To a solution of the deprotected tetrapeptide (2.56 g, 0.005 mol) in CHCl_3_ (50 mL), pentafluorophenol (1.23 g, 0.0067 mol) and EDC.HCl (0.96 g, 0.005 mol) were added followed by stirring at RT for 12 h. Filtrate of the above reaction mixture was washed with 10% NaHCO_3_ (3 × 20 mL) and 5% HCl (2 × 20 mL) solutions to obtain corresponding pentafluorophenyl ester Boc-l-prolyl-l-leucyl-l-alanyl-l-isoleucine-Opfp. Boc-group of resulting unit (2.71 g, 0.004 mol) was removed using TFA (0.91 g, 0.008 mol) and deprotected product was dissolved in CHCl_3_ (25 mL) and TEA/NMM/pyridine (2.8 mL or 2.21 mL or 1.61 mL, 0.021 mol) was added. Whole contents were then kept at 0 °C for 7 days. The reaction mixture was washed with 10% NaHCO_3_ (3 × 25 mL) and 5% HCl (2 × 25 mL) solutions. The organic layer was dried over anhydrous Na_2_SO_4_ and the crude cyclized compound was recrystallized from CHCl_3_/*n*-hexane to obtain the pure cyclic product **4**.

### 3.4. Biological Activity Studies

#### 3.4.1. Antihelmintic Screening

Newly synthesized linear and cyclic tetrapeptide **3** and **4** were subjected to antihelmintic activity studies against three different species of earthworms *Megascoplex konkanensis*, *Pontoscotex corethruses* and *Eudrilus eugeniea* at a concentration of 2 mg/mL using Garg’s method [24]. Tween 80 (0.5%) in distilled water was used as control and mebendazole was used as a standard drug. Suspensions of samples were prepared by triturating synthesized compounds (100 mg) with tween 80 (0.5%) and distilled water and the resulting mixtures were stirred using a mechanical stirrer for 30 min. The suspensions were diluted to contain 0.2% (w/v) of the test samples. Suspension of the reference drug, mebendazole, was prepared with the same concentration in a similar way. Three sets of five earthworms of almost similar sizes (2 inches in length) were placed in petri plates of 4 inch diameter containing 50 mL of suspension of test sample and reference drug at RT. Another set of five earthworms was kept as control in 50 mL suspension of distilled water and tween 80 (0.5%). The paralyzing and death times were noted and their mean was calculated for triplicate sets. The death time was ascertained by placing the earthworms in warm water (50 °C), which stimulated the movement if the worm was alive. The results of antihelmintic screening are tabulated in Table 3.

#### 3.4.2. Antimicrobial Screening

Antibacterial and antifungal screening of synthesized tetrapeptides **3** and **4** were carried out against the Gram-positive bacteria *Bacillus subtilis* and *Staphylococcus aureus*, the Gram-negative bacteria *Pseudomonas aeruginosa* and *Klebsiella pneumoniae*, dermatophytes *Microsporum audouinii* and *Trichophyton mentagrophytes*, as well as *Candida albicans* and other fungal strains, including *Aspergillus niger* at a concentration of 12.5–6 μg/mL. A modified Kirby-Bauer disc diffusion method was used for testing [25]. MIC values of test compounds were determined by tube dilution technique. Newly synthesized tetrapeptides were dissolved separately to prepare a stock solution of 1 mg/mL using DMF. Stock solution was aseptically transferred and suitably diluted with sterile broth medium to contain seven different concentrations of each test compound ranging from 200–3.1 μg/mL in different test tubes. All the tubes were inoculated with one loopful of one of the test bacteria/fungi. The process was repeated with different test bacteria/fungi and different samples. Tubes inoculated with bacterial/fungal cultures were incubated at 37 °C for 18 h and 48 h, respectively, and the presence/absence of growth of the bacteria/fungi was observed. From these results, MIC of each test compound was determined against each test bacterium/fungus. A spore suspension in sterile distilled water was prepared from 5 days old culture of the test bacteria/fungi growing on nutrient broth media/sabouraud’s broth media. About 20 mL of the growth medium was transferred into sterilized petri plates and inoculated with 1.5 mL of the spore suspension (spore concentration: 6 × 10^4^ spores/mL). Filter paper disks of 6 mm diameter and 1 mm thickness were sterilized by autoclaving at 121 °C (15 psig) for 15 min. Each petri plate was divided into five equal portions along the diameter to place one disc. Three discs of test sample were placed on three portions together with one disc with reference drug and a disk impregnated with the solvent as negative control. The petri plates inoculated with bacterial cultures were incubated at 37 °C for 18 h and those inoculated with fungal cultures were incubated at 37 °C for 48 h. Gatifloxacin and griseofulvin were used as reference drugs and DMF/DMSO were used as control. Diameters of the zones of inhibition (in mm) were measured and the average diameters for test sample were calculated for triplicate sets. The diameters obtained for the test sample were compared with that produced by the standard drugs. The results of antibacterial and antifungal studies are presented in Table 4.

## 4. Conclusions

First total synthesis of the naturally occurring tetrapeptide *cyclo*-(isoleucyl-prolyl-leucyl-alanyl) **4** was accomplished with good yield via coupling reactions utilizing carbodiimide chemistry. Pentafluorophenyl ester was proven to be effective for the activation of the acid functionality of the linear tetrapeptide unit. NMM was found to be a good base for intramolecular cyclization of the linear peptide fragment in comparison to TEA and pyridine. Synthesized cyclotetrapeptide **4** displayed potent antihelmintic activity against earthworms *M. konkanensis*, *P. corethruses* and *E. eugeniea*, along with good antifungal activity against dermatophytes *M. audouinii* and *T. mentagrophytes*, compared to the reference drugs, mebendazole and griseofulvin. In comparison, Gram-negative bacteria were found to be more sensitive than Gram-positive bacteria toward the newly synthesized peptide. On passing toxicity tests, the newly synthesized cyclooligopeptide **4** may prove to be a good candidate for clinical studies and can in the future become an anthelmintic and antidermatophyte agent.

## Figures and Tables

**Table 1:** Physical characterization data for **1**–**4**.

Compound	Physical state	M.p. (°C)	α_[D]_^a^ (°)	Yield (%)	R_f_^b^	Mol. Formula (M_r_)	Elemental analysis Calcd./found (%)		
							C	H	N
**1**	Viscous mass	-	+71.4 1	87	0.69	C_17_H_30_N_2_O_5_	59.63	8.83	8.18
						(342)	59.62	8.85	8.21
**1a**	White solid	79–80	+41.6	72	0.53	C_16_H_28_N_2_O_5_	58.52	8.59	8.53
						(328)	58.49	8.62	8.55
**2**	Viscous mass	-	−121.8	93	0.87	C_15_H_28_N_2_O_5_	56.94	8.92	8.85
						(316)	56.95	8.94	8.83
**2a**	Semisolid mass	-	−89.2	78	0.66	C_10_H_20_N_2_O_3_	55.53	9.32	12.95
						(216)	55.52	9.35	12.97
**3**	Semisolid mass	-	−55.1 2	91	0.81	C_26_H_46_N_4_O_7_	59.29	8.80	10.64
						(526)	59.26	8.79	10.66
**4**	White solid	198 (d)	−12.9 3 (−13.0)	74	0.62 *	C_20_H_34_N_4_O_4_	60.89	8.69	14.20
						(394)	60.91	8.70	14.18

a *c*, 0.5 in MeOH;

1 *c*, 0.25 in MeOH;

2 *c*, 0.15 in MeOH;

3 *c*, 0.05 in MeOH;

b (CHCl_3_:MeOH/7:3);

* (CHCl_3_:MeOH/9:1).

**Table 2:** Spectral data for **1**–**4**.

Compound	IR (CHCl_3_/KBr, *v* cm^−1^), ^1^H/^13^C NMR (CDCl_3_, *δ* ppm), ESIMS/MS (rel. int., *m/z*)
**1**	3128, 3123 (N–H str, amide), 2998–2989 (C–H str, CH_2_, Pro), 2966, 2928 (C–H str, asym, CH_3_ and CH_2_), 1748 (C=O str, ester), 1675, 1643 (C=O str, *tert* and *sec* amide), 1539 (N–H def, *sec* amide), 1391, 1377 (C–H def, *tert*-Butyl), 1384, 1369 (C–H def, *iso*-propyl), 1267 (C–O str, ester) 6.65 (1H, br. s, NH), 4.40 (1H, q, H-α, Leu), 4.05 (1H, t, *J* = 7.2 Hz, H-α, Pro), 3.62 (3H, s, OCH_3_), 3.22 (2H, t, *J* = 7.2 Hz, H-δ, Pro), 2.58 (2H, q, H-β, Pro), 1.96–1.88 (2H, m, H-γ, Pro), 1.47 (9H, s, *tert*-Butyl), 1.45–1.36 (3H, m, H-β and H-γ, Leu), 0.94 (6H, d, *J* = 6.35 Hz, H-δ, Leu)
**1a**	3297–2478 (m/br, OH str, COOH), 3125, 3122 (N–H str, amide), 2999–2992 (C–H str, CH_2_, Pro), 2968, 2929 (C–H str, asym, CH_3_ and CH_2_), 1711 (s, C=O str, COOH), 1678, 1642 (C=O str, *tert* and *sec* amide), 1536 (N–H def, *sec* amide), 1393, 1375 (C–H def, *tert*-Butyl), 1385, 1368 (C–H def, *iso*-propyl) 12.52 (1H, s, OH, COOH), 6.73 (1H, br. s, NH), 4.47 (1H, q, H-α, Leu), 3.86 (1H, t, *J* = 7.2 Hz, H-α, Pro), 3.21 (2H, t, *J* = 7.15 Hz, H-δ, Pro), 2.55 (2H, q, H-β, Pro), 1.95–1.87 (3H, m, H-γ, Pro and Leu), 1.56 (2H, t, H-β, Leu), 1.49 (9H, s, *tert*-Butyl), 0.95 (6H, d, *J* = 6.4 Hz, H-δ, Leu)
**2**	3502, 3396 (N–H str, *sec* amine), 3132, 3127 (N–H str, amide), 2965, 2928 (C–H str, asym, CH_3_ and CH_2_), 2874 (C–H str, sym, CH_3_), 1747 (C=O str, ester), 1646 (C=O str, *sec* amide), 1535 (N–H def, *sec* amide), 1269 (C–O str, ester), 1159 (C–N str, *sec* amine) 5.89 (1H, br. s, NH), 4.28 (1H, t, *J* = 6.4 Hz, H-α, Ile), 3.75–3.69 (1H, m, H-α, Ala), 3.51 (3H, s, OCH_3_), 2.03–1.95 (1H, m, H-β, Ile), 1.70–1.62 (2H, m, H-γ, Ile), 1.46 (2H, br. s, NH_2_), 1.15 (3H, d, *J* = 7.25 Hz, H-β, Ala), 0.94 (3H, t, *J* = 7.2 Hz, H-δ, Ile), 0.86 (3H, d, *J* = 6.4 Hz, H-γ′, Ile)
**3**	3129–3122 (N–H str, amide), 2999–2993 (C–H str, CH_2_, Pro), 2968, 2929, 2925 (C–H str, asym, CH_3_ and CH_2_), 2878–2873 (C–H str, sym, CH_3_), 1745 (C=O str, ester), 1676, 1646–1641 (C=O str, *tert* and *sec* amide), 1538, 1535 (N–H def, *sec* amide), 1392, 1375 (C–H def, *tert*-Butyl), 1386, 1368 (C–H def, *iso*-propyl), 1272 (C–O str, ester) 8.48 (1H, br. s, NH), 7.45 (1H, br. s, NH), 5.14 (1H, br. s, NH), 4.52 (1H, q, H-α, Leu), 4.25 (1H, q, H-α, Ala), 4.14 (1H, t, *J* = 7.2 Hz, H-α, Pro), 3.73 (1H, t, *J* = 6.35 Hz, H-α, Ile), 3.49 (3H, s, OCH_3_), 3.43 (2H, t, *J* = 7.25 Hz, H-δ, Pro), 2.49 (2H, q, H-β, Pro), 2.05–1.97 (1H, m, H-β, Ile), 1.95–1.89 (2H, m, H-γ, Pro), 1.83–1.62 (4H, m, H-β, Leu and H-γ, Ile), 1.48 (9H, s, *tert*-Butyl), 1.48–1.43 (1H, m, H-γ, Leu), 1.29 (3H, d, *J* = 7.2 Hz, H-β, Ala), 0.99 (6H, d, *J* = 6.4 Hz, H-δ, Leu), 0.93 (3H, t, *J* = 7.15 Hz, H-δ, Ile), 0.89 (3H, d, *J* = 6.45 Hz, H-γ′, Ile) 174.8 (C=O, Leu), 172.9, 172.4 (2C, C=O, Pro and Ile), 169.8 (C=O, Ala), 156.2 (C=O, Boc), 79.8 (C-α, *tert*-Butyl), 59.7 (C-α, Pro), 58.5 (C-α, Ile), 54.7 (OCH_3_), 51.3 (C-α, Ala), 49.7 (C-α, Leu), 46.9 (C-δ, Pro), 37.6, 37.1 (2C, C-β, Ile and Leu), 28.9 (C-β, Pro), 28.2 (3C, C-β, *tert*-Butyl), 24.2, 23.9 (2C, C-γ, Ile and Leu), 23.7 (C-γ, Pro), 22.3 (2C, C-δ, Leu), 17.9 (C-β, Ala), 15.1 (C-γ′, Ile), 9.5 (C-δ, Ile)
**4**	3128, 3123, 3119 (N–H str, amide), 2998, 2995–2992 (C–H str, CH_2_, Pro), 2969, 2928–2924 (C–H str, asym, CH_3_ and CH_2_), 2879, 2875 (C–H str, sym, CH_3_), 1675, 1648–1643 (C=O str, *tert* and *sec* amide), 1539–1536 (N–H def, *sec* amide), 1385, 1369 (C–H def, *iso*-propyl) 9.37 (1H, br. s, NH), 9.31 (1H, br. s, NH), 7.76 (1H, br. s, NH), 4.26 (1H, t, *J* = 7.15 Hz, H-α, Pro), 3.99 (1H, q, H-α, Ala), 3.95 (1H, q, H-α, Leu), 3.61 (1H, t, *J* = 6.4 Hz, H-α, Ile), 3.52 (2H, t, *J* = 7.3 Hz, H-δ, Pro), 2.35 (2H, q, H-β, Pro), 1.96–1.83 (2H, m, H-γ, Pro), 1.89–1.83 (1H, m, H-γ, Leu), 1.81–1.76 (1H, m, H-β, Ile), 1.73–1.64 (2H, m, H-β, Leu), 1.59–1.53 (2H, m, H-γ, Ile), 1.45 (3H, d, *J* = 7.25 Hz, H-β, Ala), 0.98 (6H, d, *J* = 6.35 Hz, H-δ, Leu), 0.95 (3H, d, *J* = 6.45 Hz, H-γ′, Ile), 0.89 (3H, t, *J* = 7.2 Hz, H-δ, Ile) 171.1 (C=O, Leu), 170.9 (C=O, Ala), 170.5, 168.1 (2C, C=O, Pro and Ile), 63.3 (C-α, Ile), 57.5 (C-α, Pro), 54.1 (C-α, Leu), 49.9 (C-α, Ala), 46.5 (C-δ, Pro), 43.8, 39.9 (2C, C-β, Leu and Ile), 29.9 (C-β, Pro), 24.8, 24.2 (2C, C-γ, Ile and Leu), 22.0 (2C, C-δ, Leu), 21.3 (C-γ, Pro), 19.2 (C-β, Ala), 15.4 (C-γ′, Ile), 9.8 (C-δ, Ile) 395.4 [(M + H)^+^, 100], 367.4 [(395.4 − CO)^+^, 11], 324.4 [(H-Ile-Pro-Leu)^+^, 32], 298.4 [(H-Leu-Ala-Ile)^+^, 39], 296.4 [(324.4 − CO)^+^, 10], 282.3 [(H-Ala-Ile-Pro)^+^, 29], 270.3 [(298.4 − CO)^+^, 17], 254.3 [(282.3 − CO)^+^, 15], 211.3 [(H-Ile-Pro)^+^, 19], 185.2 [(H-Leu-Ala)^+^, 76], 157.2 [(185.2 − CO)^+^, 12], 114.1 [(H-Leu)^+^, 19], 86.1 [Ile/Leu (C_5_H_12_N)^+^, 16], 70.1 [Pro (C_4_H_8_N)^+^, 10], 57.1 [(C_4_H_9_)^+^, 15], 44.1 [Ala (C_2_H_6_N)^+^, 12], 43.1 [(C_3_H_7_)^+^, 11], 29.1 [(C_2_H_5_)^+^, 9], 15.0 [(CH_3_)^+^, 13]

**Table 3:** Anthelmintic screening data for **3** and **4**.

Compound	Earthworm species					
	*M. konk.*		*P. core.*		*E. euge.*	

	Mean paralyzing time (min) ‡	Mean death time (min) ‡	Mean paralyzing time (min)	Mean death time (min)	Mean paralyzing time (min)	Mean death time (min)
**3**†	14.25 ± 0.42	22.57 ± 0.36	18.11 ± 0.26	29.47 ± 0.14	14.12 ± 0.23	24.54 ± 0.12
**4**†	9.28 ± 0.20	18.27 ± 0.17	12.44 ± 0.19	23.55 ± 0.27	12.40 ± 0.13	22.05 ± 0.37
Control #	-	-	-	-	-	-
Mebendazole †	13.85 ± 0.64	22.85 ± 0.53	17.82 ± 0.43	29.60 ± 0.22	13.54 ± 0.45	24.05 ± 0.62

M. konk.: Megascoplex konkanensis; P. core.: Pontoscotex corethruses; E. euge.: Eudrilus eugeniea;

† Conc. = 2 mg/mL;

‡ Data are given as mean ± S.D. (*n* = 3);

# Tween 80 (0.5%) in distilled water.

**Table 4:** Antimicrobial screening data for **3** and **4**.

Compound	Diameter of zone of inhibition (mm)							
	Bacterial strains				Fungal strains			

	*B. sub.*	*S. aur.*	*P. aeru.*	*K. pneu.*	*C. alb.*	*M. audo.*	*A. niger*	*T. menta.*
**3**	-	-	13(6) †	16(6)	12(6)	18(6)	-	19(6)
**4**	-	-	18(6)	20(6)	15(6)	23(6)	-	25(6)
Control *	-	-	-	-	-	-	-	-
Gatifloxacin	18(12.5)	28(6)	22(6)	25(6)	-	-	-	-
Griseofulvin	-	-	-	-	20(6)	17(6)	18(12.5)	20(6)

B. sub.: Bacillus subtilis; S. aur.: Staphylococcus aureus; P. aeru.: Pseudomonas aeruginosa; K. pneu.: Klebsiella pneumoniae; C. alb.: Candida albicans; M. audo.: Microsporum audouinii; A. niger: Aspergillus niger; T. menta.: Trichophyton mentagrophytes;

† Values in bracket are MIC values (μg/mL);

* DMF/DMSO.
